# Thermal Characteristics of Staggered Double-Layer Microchannel Heat Sink

**DOI:** 10.3390/e20070537

**Published:** 2018-07-19

**Authors:** Dalei Jing, Lei He

**Affiliations:** School of Mechanical Engineering, University of Shanghai for Science and Technology, Shanghai 200093, China

**Keywords:** double-layered microchannel heat sink, thermal resistance, microchannel aspect ratio

## Abstract

The present work numerically studies the thermal characteristics of a staggered double-layer microchannel heat sink (DLMCHS) with an offset between the upper layer of microchannels and lower layer of microchannels in the width direction, and investigates effects of inlet velocity and geometric parameters including the offset of the two layers of microchannels, vertical rib thickness and microchannel aspect ratio on the thermal resistance of the staggered DLMCHS. The present work found that the thermal resistance of the staggered DLMCHS increases with the increasing offset value when the vertical rib thickness is small, but decreases firstly and then increases as the offset value increases when the vertical rib thickness is large enough. Furthermore, the thermal resistance of the staggered DLMCHS decreases with the increasing offset when the aspect ratio is small, but increases with the increasing offset when the aspect ratio is large enough. Thus, for the DLMCHS with a small microchannel aspect ratio and large vertical rib thickness, the offset between the upper layer of microchannels and the lower layer of microchannels in the width direction is a potential method to reduce thermal resistance and improve the thermal performance of the DLMCHS.

## 1. Introduction

With the development of miniaturization and integration of micro-electro-mechanical systems (MEMS), micro-electro-mechanical devices like microelectronic chips are faced with a severe challenge of high power density and the resulting high working temperature, which can significantly affect their performances and life span. Thus, cooling techniques with excellent heat dissipation performances are urgently desired. Single-layer microchannel heat sink (SLMCHS) first introduced by Tuckerman and Pease [1] was found to be one of the most excellent cooling techniques to remove high heat flux for microelectronic chips due to its advantages including small size, compactness and low cost; and it has been extensively used for the cooling of microelectronic devices.

The heat and mass transfer performances of the SLMCHS have been studied and optimally designed from various aspects such as geometric size, cross-sectional shape of microchannel, microchannel layout of the heat sink and interfacial properties [2,3,4,5,6,7,8,9,10,11,12,13,14,15]. Although SLMCHS can effectively remove excessive heat, it presents the disadvantage of large temperature variation, and then results in thermal stress in the devices and reduces the performances of the devices. A feasible method to reduce the large temperature variation faced by SLMCHS is by enhancing the coolant flow rate at the cost of more pumping power, however, it is also accompanied with the problems of bulky packages and more noise. To solve these problems, Vafai and Zhu [16] first developed the double-layered microchannel heat sink (DLMCHS). Followed by the work of Vafai and Zhu, comprehensive studies have been performed to investigate the flow and thermal characteristics of the DLMCHS. For example, Chong et al. [17] investigated and compared the thermal performances of a single layer counter flow heat sink and a double layer counter flow microchannel heat sink with rectangular channels using the thermal resistance approach. Hung et al. [18] numerically investigated the heat transfer characteristics of a DLMCHS and analyzed the effects of substrate materials, coolants, channel number, channel width ratio, channel aspect ratio, substrate thickness and pumping power on the temperature distribution, pressure drop, and thermal resistance of the DLMCHS. Wong et al. [19] numerically studied and compared the heat transfer performances of a DLMCHS both in parallel flow and counter flow configurations, and they found that when the channel aspect ratio and Reynolds number are low, the case of parallel flow can show better thermal performance over the case of counter flow. Wu et al. [20] conducted a numerical parametric study on the performances of DLMCHS and investigated the effects of channel width ratio, aspect ratio and velocity ratio on the thermal resistance of the DLMCHS. Wei et al. [21] experimentally and numerically studied the effects of flow direction in counter flow or parallel flow, flow rate allocation among layers, and non-uniform heating on the thermal performance of DLMCHS. Additionally, optimization design of the DLMCHS has also been carried out to achieve better heat and mass transfer performances. For example, Hung et al. [22] analyzed the optimization geometric parameters including number of channels, channel width ratio and channel aspect ratio of DLMCHS to achieve a minimum thermal resistance under a given bottom area and heat flux. Lin et al. [23] conducted optimization design of DLMCHS to obtain minimum global thermal resistance. Six design variables including channel number, bottom channel height, vertical rib width, thicknesses of two horizontal ribs, and coolant velocity in the bottom channel were simultaneously optimized under the different constraints of a fixed pumping power, coolant volumetric flow rate or pressure drops through the DLMCHS. Leng et al. [24] performed the optimization of channel number, channel width, bottom channel height, and bottom coolant inlet velocity to achieve the optimal heat sink performances of thermal resistance and bottom wall temperature uniformity for DLMCHS under the given pumping power. They [25] also performed a multi-parameter optimization of a new DLMCHS with truncated top channels, and channel number, channel height and channel width and the dimensionless truncation length of top channels were optimized to achieve a minimum overall heat resistance at various fixed pumping powers and fixed coolant volumetric flow rates.

From the above literatures, it can be found that although there are extensive studies on the effects of different parameters, such as the channel geometric sizes, channel shape, channel layout and layers of channel on the flow and thermal performances of the MCHS, relevant studies are still needed to further reach optimal heat and mass performances. Based on the thermal resistance network model, the total thermal resistance of a MCHS is consisting of the conduction resistance of the base, the conduction resistance of vertical rib, the convective resistance from the base to liquid, the convective resistance from the vertical rib to liquid, and the convective resistance of the liquid. Among these resistances, the conduction resistances of the base and the vertical rib are directly related to the thickness of the vertical rib. For a DLMCHS, when there is an offset between the two layers of microchannels in the width direction, the conduction resistance of the base or the vertical rib on one side increases, however, the conduction resistance on the other side decreases. The overall effect of the offset on the thermal resistance is unknown, and it may be a potential method to optimize the total thermal resistance. Based on the above consideration, the present work proposes a staggered DLMCHS with offset in the width direction, numerically investigates the thermal characteristics of the staggered DLMCHS and analyzes the effects of geometric parameters including offset of the two layers of microchannels, vertical rib thickness and aspect ratio of the microchannel on the thermal resistance of the staggered DLMCHS.

## 2. Numerical Analysis

### 2.1. Description of the Staggered DLMCHS

**Figure 1:**
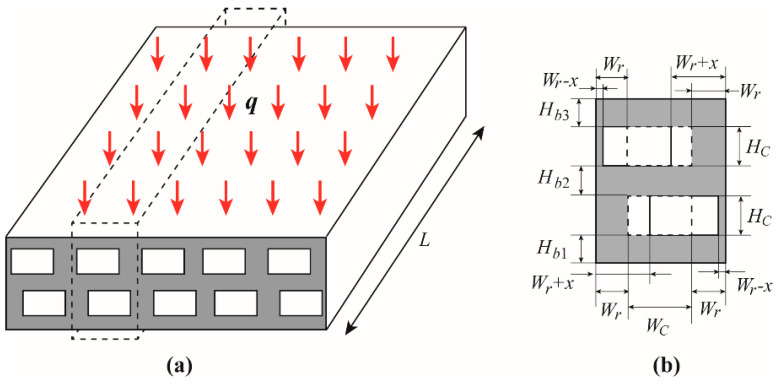
(**a**) Schematic of a staggered DLMCHS and the dashed line frame gives the repeated computational domain; (**b**) cross section of the repeated computational domain.

Figure 1 gives the schematic of a staggered DLMCHS. Each layer of the staggered DLMCHS has *N* microchannels and *N* + 1 vertical ribs. Considering the manufacturability, each microchannel of the staggered DLMCHS is in rectangle shape. The staggered DLMCHS exposes to a constant heat flux through the top plate and the remaining surfaces of the heat sink are assumed to be thermally insulated; coolant is driven to through the two layers of microchannels to remove the heat. Both the parallel flow and counter flow are considered in the present work. Considering the symmetry of the heat sink, only the computational domain limited by the dash line frame in Figure 1 is analyzed. Its geometric parameters of the repeated computational domain are as following. The length of the computational domain is fixed at length *L* = 30 mm. The channels at the upper and lower layers have the same channel width *W_C_* and height *H_C_*. The horizontal ribs at the top, middle and base have the same thickness *H_b_*_1_ = *H_b_*_2_ = *H_b_*_3_. *W_r_* is the vertical rib thickness when both the upper channel and the lower channel locate at the centerline of the computational domain. *x* is the offset in the width direction of both the upper channel and the lower channel to the opposite directions. Additionally, in order to analyze the effects of aspect ratio of the microchannel on the thermal characteristics of the staggered DLMCHS, the width and height of each microchannel is adjusted under the constraint of a constant channel cross-sectional area of 0.4 mm × mm.

### 2.2. Governing Equations

Assume that (1) the fluid flow and heat transfer in the staggered DLMCHS are in steady-state; (2) the fluid flow is incompressible, single phase, and laminar flow; (3) effects of gravity and other forms of body forces are negligible, then, the fluid flow and heat transfer in the staggered DLMCHS can be governed by the following Continuity, Momentum and Energy equations

Continuity equation:

(1) $$\;\nabla \vec{v}=0\;$$

Momentum equation:

(2) $$\;\rho \left(\vec{v}\cdot \nabla \vec{v}\right)=-\nabla p+\nabla \cdot \left(\mu \nabla \vec{v}\right)\;$$

Energy equation:

(3) $$\;\{\begin{array}{c} \rho c_{p}\left(\vec{v}\cdot \nabla T\right)=k_{f}\nabla^{2}T\;\;\;\;\;\;\;\mathrm{in}\;\mathrm{fluid}\;\mathrm{flow}\\ k_{s}\nabla^{2}T_{s}=0\;\;\;\;\;\;\;\;\;\;\;\;\;\;\;\;\mathrm{in}\;\mathrm{solid} \end{array}\;$$

where *v* is the velocity field of fluid flow, *ρ* is the density of the liquid, *p* is the pressure and *μ* is the dynamic viscosity of the liquid, *c_p_* is the specific heat of the liquid, *k_f_* is the thermal conductivity of the liquid, *T* is the temperature field in the liquid, *k_s_* is the thermal conductivity of the solid heat sink and *T_s_* is the temperature field in the solid heat sink.

The initial conditions and boundary conditions for the equations governing the fluid flow and heat transfer are given as,

(4) $$\;\{\begin{array}{c} v=v_{in},T=T_{in}=298.15\;K\;\;\;\;\mathrm{at}\;\mathrm{the}\;\mathrm{inlet}\;\\ p=p_{out}\;\;\;\;\;\;\mathrm{at}\;\mathrm{the}\;\mathrm{outlet}\\ \;v_{w}=0,T=T_{s},-k_{f}\left(\frac{\partial T}{\partial n}\right)=-k_{s}\left(\frac{\partial T_{s}}{\partial n}\right)\;\;\;\mathrm{at}\;\mathrm{the}\;\mathrm{wall}\\ q=10W/{\mathrm{cm}}^{2}\;\;\;\mathrm{at}\;\mathrm{the}\;\mathrm{top}\;\mathrm{plate} \end{array}\;$$

where *v_in_* and *T_in_* are the inlet velocity and inlet temperature of the fluid, respectively, *p_out_* is the outlet pressure of the fluid, *v_w_* is the velocity at the solid-liquid interface and its zero value reflects the no-slip boundary condition, *n* is the local coordinate normal to the wall, and *q* is the heat flux applied on the top plate of the heat sink.

Considering deionized water has a high heat capacity and thermal conductivity, it displays good heat transfer performances, thus, deionized water is chosen as the cooling liquid, and the solid heat sink is in the material of silicon. The physical properties of water are temperature dependent, however, the physical properties of silicon heat sink are assumed to be temperature independent with fixed values of *k_s_* = 130 W/(m × K), *c_ps_* = 700 J/(kg × K) and *ρ_s_* = 2329 kg/m^3^. This is reasonable for the present work because the temperature variation caused by the heat flux of 10 W/cm^2^ within the silicon heat sink is relatively small and the corresponding variation in thermophysical properties of silicon is small enough to be neglectable. However, when the heat flux is large enough up to hundreds even thousands of W/cm^2^, the temperature-dependent thermophysical properties of silicon must be considered.

The present work focuses on the overall heat transfer performance of the DLMCHS, including the heat conduction of the solid heat sink and heat convection of the coolant. Correspondingly, the total thermal resistance of the staggered DLMCHS is defined as following to evaluate its heat transfer performance of the staggered DLMCHS [18,19,22,23,24,25].

(5) $$\;R_{T}=\frac{T_{max}-T_{in}}{qA}\;$$

where *R_T_* is the total thermal resistance of the staggered DLMCHS, *T_max_* is the maximum temperature of the heated wall along the centerline of the fluid–solid interface, and *T_in_* is the inlet temperature of the coolant, and *A* is the area of the top plate of the heat sink.

## 3. Results and Discussion

**Figure 2:**
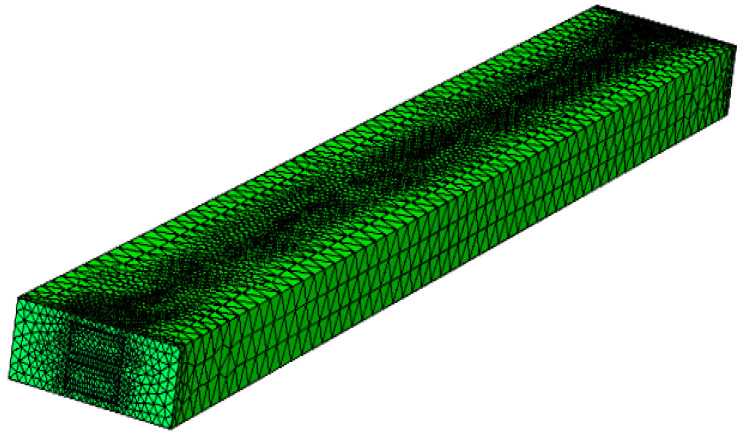
Example of computational grid used in present work.

In the present work, COMSOL 5.3 is used to carry out the numerical analyses to study the thermal characteristics of the staggered DLMCHS. Meanwhile, the grid sensitivity test is carefully performed to keep the accuracy of the numerical analysis. An example of the grid sensitivity test is as following. Four different numbers of tetrahedral meshes are used during the grid sensitivity test: 5.7 × 10^4^, 1.4 × 10^5^, 2.7 × 10^5^ and 3.2 × 10^5^, and the corresponding maximum temperature and the relative errors of the maximum temperature relating to the mesh number are given in Table 1. Then, comprehensive considering the simulation efficiency and accuracy, the numerical analysis with 2.7 × 10^5^ meshes is chosen and Figure 2 gives an example of the computational grid used in the present work.

**Figure 3:**
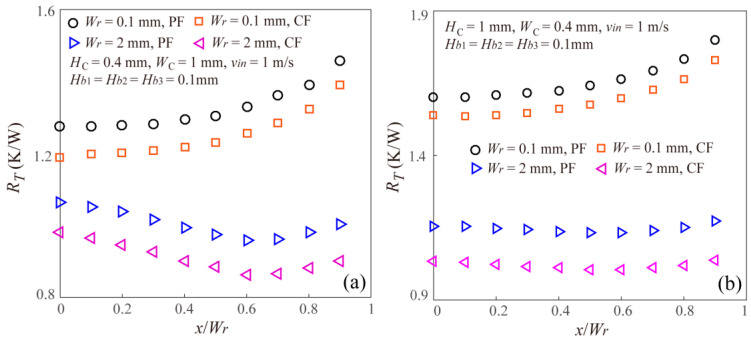

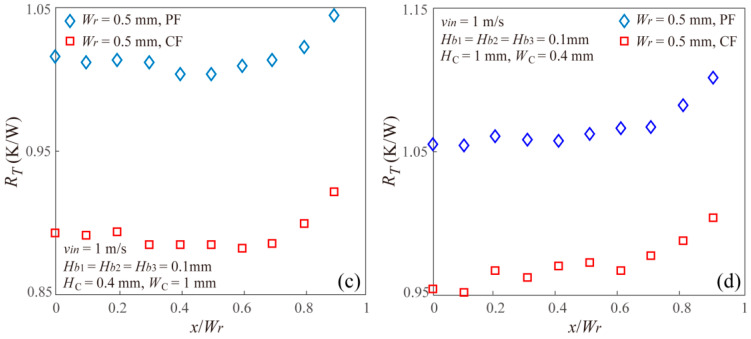
Effects of offset on the thermal resistance of the staggered DLMCHS with different microchannel aspect ratios and vertical rib thicknesses for both parallel flow and counter flow. (**a**) *H_C_* = 0.4 mm, *W_C_* = 1 mm and *W_r_* = 0.1 mm and 2 mm; (**b**) *H_C_* = 1 mm, *W_C_* = 0.4 mm and *W_r_* = 0.1 mm and 2 mm; (**c**) *H_C_* = 0.4 mm, *W_C_* = 1 mm and *W_r_* = 0.5 mm; (**d**) *H_C_* = 1 mm, *W_C_* = 0.4 mm and *W_r_* = 0.5 mm.

Based on the numerical analysis, Figure 3 gives the effects of offset on the thermal resistance of the staggered DLMCHS with different microchannel aspect ratios and different vertical rib thicknesses. From the results showing in Figure 3, it can be found there are three different variation trends of thermal resistance with the offset in the width direction both for the parallel flow (PF) and counter flow (CF). When the vertical rib thickness is small enough, for example *W_r_* = 0.1 mm, the thermal resistance of the staggered DLMCHS shows an increasing trend with the increasing offset, which means the conventional DLMCHS with zero offset has the smallest thermal resistance and shows the best thermal performance. However, when the vertical rib thickness is large enough, for example *W_r_* = 2 mm in the present work, the thermal resistance of the staggered DLMCHS decreases firstly and then increases as the offset increases, which means there is an optimal offset value for the staggered DLMCHS to achieve a minimum thermal resistance and improve the thermal performance. When the vertical rib thickness has an intermediate value, for example *W_r_* = 0.5 mm, the thermal resistance of the staggered DLMCHS shows a much more complicated increasing trend in fluctuation. This means that shift between the upper microchannel and the lower microchannel in the width direction is a potential method to reduce thermal resistance and improve the thermal performance of the DLMCHS, especially for the DLMCHS with large vertical rib thickness.

Additionally, it can be found that for all the staggered DLMCHS with the same geometry dimensions, the case of counter flow shows a smaller thermal resistance than the case of parallel flow, which is consistent with previous studies [19,21].

**Figure 4:**
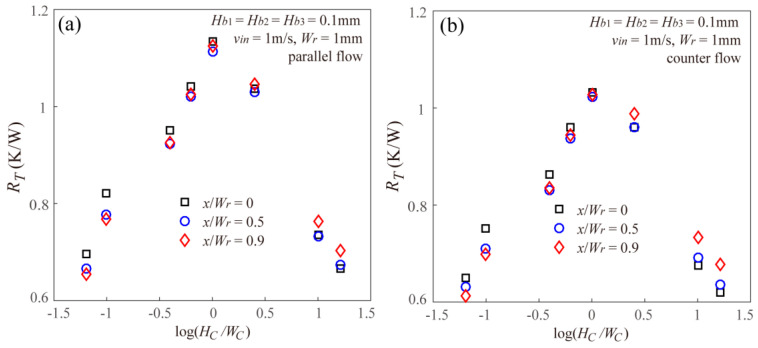
Effects of microchannel aspect ratio on the thermal resistance of the staggered DLMCHS with different offset values for both (**a**) parallel flow and (**b**) counter flow.

Figure 4 gives the effect of microchannel aspect ratio on the thermal resistance of the staggered DLMCHS with different offset and the same vertical rib thickness for both parallel flow and counter flow. From the results showing in Figure 4, it can be found that under the constraint of a constant channel cross-sectional area, the thermal resistance of the staggered DLMCHS shows a decreasing after an increasing trend with the increasing microchannel aspect ratio. When the microchannel aspect ratio is one, that is, the staggered DLMCHS with the square-shaped microchannel has the largest thermal resistance. This is because under the constraint of a constant channel cross-sectional area, the area of the solid-liquid interfaces of the microchannel for cooling shows an increasing after decreasing trend as the aspect ratio increases, and the square shaped microchannel with the aspect ratio being equal to one has the smallest cooling area. The microchannel with a larger cooling area can remove more heat applied at the top plate and results in a smaller thermal resistance.

More important, it can be found from Figure 4 that the microchannel aspect ratio can make significant and totally different effects on the thermal resistance of the staggered DLMCHS with the fixed vertical rib thickness and different offset. Both for the parallel flow and counter flow, the thermal resistance of the staggered DLMCHS decreases with the increasing offset when the aspect ratio is small enough, but increases with the increasing offset when the aspect ratio is large enough for the three values of offset. This means that for the DLMCHS with the small microchannel aspect ratio, it is much easier for the offset between the upper layer and the lower layer to reduce the thermal resistance and improve the thermal performances of the DLMCHS.

**Figure 5:**
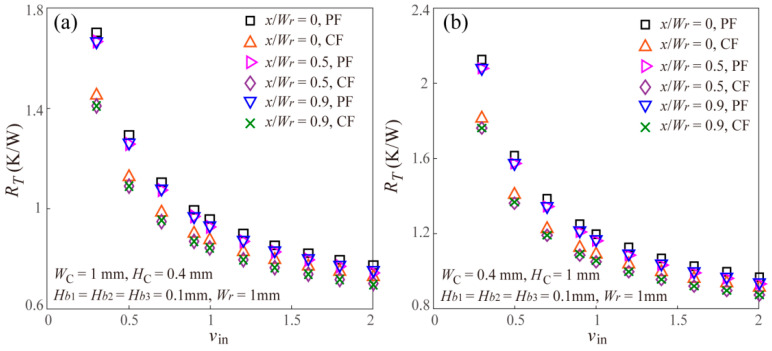
Effects of inlet velocity on the thermal resistance of the staggered DLMCHS with different offset values and channel dimensions of (**a**) *W_C_* = 1 mm, *H_C_* = 0.4 mm and (**b**) *W_C_* = 0.4 mm, *H_C_* = 1 mm for both parallel flow and counter flow.

Additionally, Figure 5 gives the effect of inlet velocity of the coolant on the thermal resistance of the staggered DLMCHS with different offset and the same vertical rib thickness for both parallel flow and counter flow. It should be noted that being different from some previous studies displaying the effect of Reynolds number on the thermal performances of the MCHS [5,7,19], the properties of the coolant of water including density and dynamic viscosity are dependent on the temperature in the present work, thus, it is difficult to accurately calculate the Reynolds number when the inlet velocity varies, thus, the present work gives the effect of inlet velocity on the thermal resistance in Figure 5. From Figure 5, it can be found that the thermal resistance of the staggered DLMCHS decreases with the increasing inlet velocity. This is easy to be understood as follows. A larger inlet velocity means a larger flow rate, and will remove much more heat and reduce the thermal resistance of the staggered DLMCHS. This result regarding the effect of inlet velocity on the thermal resistance of the staggered DLMCHS is consistent with the previous studies [20,21].

## 4. Conclusions

In the present work, a staggered DLMCHS with an offset in the width direction between the upper layer of microchannels and lower layer of microchannels was proposed, and its thermal resistance was numerically investigated. Furthermore, effects of inlet velocity and geometric parameters including offset of the two layers of microchannels, vertical rib thickness and microchannel aspect ratio on the thermal resistance of the staggered DLMCHS were analyzed. The conclusions of the present work are as following.

(1) The thermal resistance of the staggered DLMCHS increases with the increasing offset value when the vertical rib thickness is small enough, but decreases firstly and then increases as the offset value increases when the vertical rib thickness is large enough, which means there is an optimal offset value for the staggered DLMCHS to achieve a minimum thermal resistance when the vertical rib thickness is large enough.
(2) The thermal resistance of the staggered DLMCHS shows a decreasing after increasing trend with the increasing microchannel aspect ratio under the constraint of a constant channel cross-sectional area. More important, the thermal resistance of the staggered DLMCHS decreases with the increasing offset when the aspect ratio is small enough, but increases with the increasing offset when the aspect ratio is large enough. This means that for the DLMCHS with a small microchannel aspect ratio, it is much easier for the offset between the upper layer and the lower layer to reduce the thermal resistance and improve the thermal performances of the DLMCHS.
(3) The thermal resistance of the staggered DLMCHS decreases with the increasing inlet velocity.

The present work shows that a shift between the upper layer of microchannels and the lower layer of microchannels in the width direction is a potential method to reduce thermal resistance and improve the thermal performance of the DLMCHS, however, this improvement is highly dependent on the geometric parameters of the DLMCHS including vertical rib thickness and microchannel aspect ratio.

## Figures and Tables

**Table 1:** Example of grid sensitivity test for the numerical analysis of staggered DLMCHS.

Test Number *i*	Number of Tetrahedral Mesh	*T_max_* [°C]	(*T_max_^i^*^+1^ − *T_max_^i^*)/*T_max_^i^*
0	5.7 × 10^4^	35.296	-
1	1.4 × 10^5^	32.862	6.9 × 10^−2^
2	2.7 × 10^5^	32.458	1.2 × 10^−2^
3	3.2 × 10^5^	32.411	1.5 × 10^−3^
